# Editorial: Neuropsychological mechanism of psychological resilience in relation to mental health outcomes across the lifespan

**DOI:** 10.3389/fpsyt.2023.1242182

**Published:** 2023-06-28

**Authors:** Bess Yin-Hung Lam

**Affiliations:** Department of Counselling and Psychology, Hong Kong Shue Yan University, North Point, Hong Kong SAR, China

**Keywords:** resilience, psychological factors, family factors, scale validation, underlying mechanism, mental health

In face of the COVID-19 pandemic which has lasted for more than 2 years affecting all aspects of our lives across the world, psychological resilience has become an especially important trait in everyone one of us for better adaptation in such a difficult time. According to the American Psychological Association Dictionary of Psychology (1), it is defined as “the process of adapting well in the face of adversity, trauma, tragedy, threats, or significant sources of stress, or “bouncing back” from difficult experiences.” Along with the same line, previous study showed that better adaptation to chronic illness and traumatic disabilities was found in those with higher resilience. For instance, Lam et al. (2) examined whether psychological resilience can be a protective factor among older adults with mild cognitive impairment (MCI). Results showed that higher level of resilience in addition to the conventional cognitive reserve proxies predicted lower MCI risks. In addition, MCI participants who were more resilient had better visuospatial ability than their lower level counterparts. These findings call for the importance of future research addressing the level of resilience in older adults for healthy aging. Although prior studies have reported that psychological resilience is associated with better mental outcomes such as reduced psychosis and dementia symptoms, the underlying mechanism warrants further investigation.

Given there is an emerging need for investigating the neuropsychological correlates of this important psychological construct, the manuscripts published under this Research Topic aim to explore the neuropsychological mechanism of psychological resilience in individuals across age groups. Specifically, three of those studies have examined different psychological underpinnings ranging from social-cognitive to family factors that are associated with psychological resilience in healthy college students (Chen et al.), adults living in the pandemic time (Skalski et al.), fathers and mothers of patients with cleft lip and/or palate (Yuan et al.). On the other hand, Chow et al. reported the findings to support the development and validation of Family Resilience Scale Short Form (FRS16). It is also noteworthy that FRS16 has taken a broader perspective by tapping on family resilience instead of individual resilience which has been the focus in the literature. These findings can potentially help mitigate and inform clinical practices to enhance psychological resilience, thereby preventing mental health problems across the lifespan.

Regarding the factors associated with resilience reported in this Research Topic, Chen et al. found that the association between adverse childhood experiences and psychological wellbeing was mediated by psychological resilience and this mediated relationship was different across gender. Specifically, resilience was negatively related to abuse/neglect, which in turn had a negative association with mental well-being specifically in female participants while household challenges were inversely associated with psychological well-being via reduced resilience in males. These findings suggest that gender and resilience are both important factors of psychological wellbeing in those with risk factors such as adverse childhood experiences. Furthermore, another study investigated psychological resilience in the parents of patients with clef lip and/or palate who are at risk for poor psychological wellbeing (Yuan et al.). Results showed that hope was the only communal variable strongly associated with resilience among both the fathers and the mothers. Moreover, a few differential correlates of psychological resilience were found in fathers (e.g. job status and medical payments) and mothers (e.g., perceived social support and the age of patients), respectively. These findings suggest that enhancing hope in parents of patients with cleft lip and/or palate might help enhance their resilience and there is a need to develop specific intervention for fathers and mothers to prompt their resilience. Not only that resilience plays an important role in the psychological wellbeing of people who have childhood and familial risk factors, Skalski et al. found that persistent thinking and anxiety related to the pandemic acted as a mediator of the relationship between resilience and psychological wellbeing in healthy adults. These findings suggest that persistent thinking and anxiety related to prolonged stressors as well as psychological resilience are key factors that should also be addressed by practitioners in interventions so to enhance one's mental health especially during the pandemic or the spread of infection diseases.

These findings published in this Research Topic should only be the beginning to the investigation of underlying mechanism of psychological resilience. Three of those studies investigated Chinese participants while Chow et al. also examined participants from the US. These findings provide much insight to the field, which has been focusing more on the western population in prior literature, in understanding the construct of psychological resilience in Chinese or Asian population. Nevertheless, much more light should be shed on this topic in future studies for empowering and enhancing psychological resilience, thereby mental wellbeing in the community. In addition to psychosocial underpinnings, the neural basis of psychological resilience should be further addressed in future studies. Furthermore, cultural and age factors should also be examined to help develop age and culturally appropriate prevention and interventions to enhance resilience in the future. Last but not least, in addition to individual resilience, different types of resilience including family and community resilience warrants much more investigation in order to help develop a better understanding of the holistic impact of resilience at different levels.

## Author contributions

The author confirms being the sole contributor of this work and has approved it for publication.
